# Dynamics of Antibody Response to BNT162b2 mRNA COVID-19 Vaccine: A 7-Month Follow-Up Study

**DOI:** 10.3390/medicina57121330

**Published:** 2021-12-05

**Authors:** Tudor Rares Olariu, Sorin Ursoniu, Iosif Marincu, Maria Alina Lupu

**Affiliations:** 1 Discipline of Parasitology, Department of Infectious Disease, Victor Babes University of Medicine and Pharmacy, 300041 Timisoara, Romania; imarincu@umft.ro; 2 Clinical Laboratory, Municipal Clinical Emergency Teaching Hospital, 300041 Timisoara, Romania; 3 Center for Diagnosis and Study of Parasitic Diseases, Department of Infectious Disease, Victor Babes University of Medicine and Pharmacy, 300041 Timisoara, Romania; 4 Center for Translational Research and Systems Medicine, Department of Functional Sciences, Victor Babes University of Medicine and Pharmacy, 300041 Timisoara, Romania; sursoniu@yahoo.com; 5 Clinical Laboratory, Institute of Cardiovascular Diseases, 300041 Timisoara, Romania

**Keywords:** SARS-CoV-2, antibodies, spike protein, COVID-19, vaccine, healthcare workers, Romania

## Abstract

*Background and Objectives:* Comprehension regarding immunity to severe acute respiratory syndrome coronavirus 2 (SARS-CoV-2) is limited, and the durability of immune responses after vaccination is currently unknown. Several studies have reported on the antibody response in fully vaccinated individuals with a limited follow-up of the participants, i.e., below 7 months. *Materials and Methods:* The antibody response to complete vaccination with the BNT162b2 mRNA COVID-19 vaccine was assessed monthly, for 7 months, in 92 healthcare workers, between February 26 and September 26, 2021. The SARS-CoV-2 anti-spike protein IgG (IgG_S_) antibody was detected using the SARS-CoV-2 IgG II Quant assay (Abbott, Diagnostics Division, Sligo, Ireland), a chemiluminescent microparticle immunoassay (CMIA) with a sensitivity of 98.1% and specificity of 99.6%. Participants were divided into two groups, one for individuals previously infected with SARS-CoV-2 and the other for individuals without previous infection. *Results:* The median IgG_S_ titers decreased monthly both in previously infected individuals and in the uninfected group. Previously infected individuals had significantly higher median titers of IgG_S_ compared with previously uninfected subjects at all seven time points after complete vaccination (*p* < 0.001). *Conclusions:* Seven months after vaccination, the median IgG_S_ titer had decreased by more than 92% both in individuals previously infected with SARS-CoV-2 and in uninfected individuals. However, IgG_S_ antibodies were still detected in all study participants and persisted throughout the 7 months after the second dose of the vaccine. Further studies should be conducted to monitor the antibody response to the BNT162b2 mRNA vaccine beyond 7 months, to assess the need for a new booster dose in order to extend the duration and amplitude of the specific immune response.

## 1. Introduction

Full immunization with two doses of the BNT162b2 mRNA COVID-19 vaccine (Pfizer-BioNTech) has been shown to have a consistent high efficacy (95%) [1,2], regardless of age, sex, race, or associated pathology, with or without evidence of previous severe acute respiratory syndrome coronavirus 2 (SARS-CoV-2) infection [3]. Vaccination success can now be confirmed based on the detection of specific antibodies against the receptor-binding domain of the S1 subunit of the spike protein, by using different assays: electrochemiluminescence sandwich immunoassay (ECLIA), chemiluminescence microparticle immunoassay (CMIA), chemiluminescence immunoassay (CLIA), or enzyme-linked immunosorbent assay (ELISA) [4]. However, comprehension regarding immunity to SARS-CoV-2 is limited [5], and the durability of immune responses after vaccination is currently unknown [6]. Several studies have reported on the antibody response in fully vaccinated individuals with a limited follow-up of the participants, i.e., below 7 months [7,8,9]. To evaluate the antibody kinetics in individuals vaccinated with a two-dose regimen of the BNT162b2 vaccine, a longer follow-up period is needed. This is important, especially to assess the need for a new booster dose in order to extend the duration and amplitude of the specific immune response.

In this study, we evaluated the SARS-CoV-2 antibody response in healthcare workers who were vaccinated with the Pfizer-BioNTech mRNA vaccine two-dose regimen in Western Romania. The antibody response to the BNT162b2 mRNA COVID-19 vaccine was assessed monthly, for 7 months, in two groups: individuals previously infected with SARS-CoV-2 and individuals without previous infection.

## 2. Materials and Methods

### 2.1. Study Design and Population

Ninety-seven fully vaccinated volunteers, residents of Timis County (705,113 inhabitants), Romania, were initially enrolled in this study. The individuals were all healthcare workers from the Municipal and County Clinical Emergency Teaching Hospitals in Timisoara, Romania. Five study participants (3 males and 2 females) were infected with SARS-CoV-2 in the first month after vaccination, confirmed by real-time polymerase chain reaction (RT-PCR), and were excluded from this study. Finally, 92 people were included in the study. Study participants were healthy adults, with no symptoms possibly related to infection in the 14 days prior to enrollment in the study.

Of the 92 subjects, 33 had confirmed SARS-CoV-2 past infection, by RT-PCR and/or detection of SARS-CoV-2 anti-nucleocapsid (N) protein IgG (IgG_N_), and 59 subjects had not been infected with SARS-CoV-2. Previously SARS-CoV-2 infected and uninfected individuals were grouped into two age categories (24–44 years and 45–66 years).

Serum samples of the 92 individuals were collected between February 26, 2021 and September 26, 2021. All study participants had been vaccinated with 2 doses of the BNT162b2 mRNA COVID-19 vaccine (Pfizer-BioNTech, Mainz, Germany), 21 days apart [1]. Each participant was tested monthly for 7 months, starting at 1 month after receiving the second dose of the vaccine. Samples were kept at –20 °C until laboratory tests were performed at the Clinical Laboratory of the Municipal Clinical Emergency Hospital in Timisoara, a reference laboratory for COVID-19 testing in Romania.

Informed consent was obtained from all subjects involved in the study. The Ethics Committee of the Municipal Clinical Emergency Teaching Hospital in Timisoara, Romania approved this study.

### 2.2. Serologic Tests

The vaccine-elicited antibody response, SARS-CoV-2 anti-spike (S) protein IgG (IgG_S_), was detected using the SARS-CoV-2 IgG II Quant assay (Abbott, Diagnostics Division, Sligo, Ireland), a chemiluminescent microparticle immunoassay (CMIA) with a sensitivity of 98.1% and specificity of 99.6% [10]. The assay was performed on the Abbott Alinity i system (Abbott Laboratories, Lake Bluff, IL, USA). Serum samples with cutoff ≥50.0 AU/mL were considered positive for IgG_S_ [10].

To detect previous SARS-CoV-2 infections, serum SARS-CoV-2 anti-nucleocapsid (N) protein IgG (IgG_N_) was detected using the SARS-CoV-2 IgG_N_ assay (Abbott, Diagnostics Division, Sligo, Ireland), a CMIA with a sensitivity of 100% and specificity of 99% [9]. The assay was performed on the Abbott Alinity i system (Abbott Laboratories, Lake Bluff, IL, USA). Serum samples were considered positive at cutoff index ≥1.4 [10].

### 2.3. PCR Test

Real-time RT-PCR was performed on the Bio-Rad CFX96 Touch Real-Time PCR Detection System (Bio-Rad, Feldkirchen, Germany) using the FTD™ SARS-CoV-2 Kit/32 tests by Siemens Healthineers (Siemens Healthcare GmbH) with 100% positive percent agreement and 100% negative percent agreement between FTD SARS-CoV-2 and the Food and Drug Administration (FDA) authorized RT-PCR test for the detection of SARS-CoV-2 in nasopharyngeal swabs [11].

Serologic and PCR test kits were used according to the protocol specified by the manufacturer (including controls), and interpretation of the results was based on the manufacturer’s criteria.

### 2.4. Statistical Analysis

Data were compiled in a Microsoft Excel database, version 2011 (Microsoft Corp, Redmond, WA, USA). Statistical analyses were conducted with Epi Info Version 7.2 (CDC, Atlanta, GA, USA) and Stata 16.1 (Statacorp, Texas, USA). Data are presented as means ± standard deviations, medians, and interquartile range (IQR). We used two-sided Wilcoxon tests, without adjustment for multiple testing to evaluate the differences between previously SARS-CoV-2 infected and uninfected vaccinees with respect to different characteristics. A probability level of *p* < 0.05 was considered to indicate statistical significance.

## 3. Results

### 3.1. Study Participants

The 92 healthcare workers enrolled in the study were aged between 24 and 66 years (mean age = 46.57 ± 10.51 years). Twenty-seven participants (29.35%) were males, and 36 participants (39.13%) were aged 24–44 years. Female participants were aged 24–65 years (mean age = 45.97 ± 9.79 years), and males were aged 26–66 years (mean age = 48.04 ± 12.15).

#### 3.1.1. SARS-CoV-2 Previously Infected Participants

The 33 study participants with confirmed SARS-CoV-2 past infection were aged 24–65 years (mean age = 46.75 ± 11.83 years). Twelve (36.36%) were males, and 13 (39.39%) were aged 24–44 years.

#### 3.1.2. Uninfected Participants

The 59 individuals who had not been previously infected with SARS-CoV-2 were aged 26–66 years (mean age = 46.47 ± 9.80 years). Fifteen (25.42%) were males, and 23 (38.98%) were aged 24–44 years.

### 3.2. Antibody Response to BNT162b2 mRNA COVID-19 Vaccine

**Figure 1:**
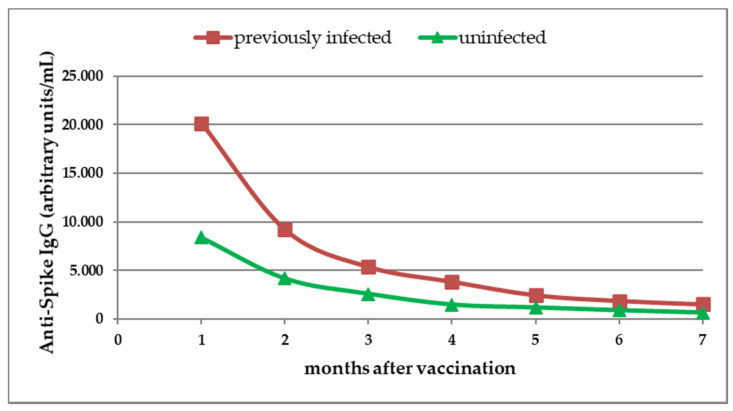
Dynamics of SARS-CoV-2 anti-spike (S) protein IgG (IgG_S_) antibodies after complete vaccination. Antibody titers were evaluated monthly for 7 months. Median titers of IgG_S_ antibodies were assessed in previously infected individuals and in uninfected individuals.

Circulating IgG_S_ antibodies were detected in all study participants and persisted throughout the 7 months after the second dose of the vaccine. Our results revealed a monthly reduction in median IgG_S_ titer, and the decreasing trend of antibody response was seen in both SARS-CoV-2 previously infected individuals and in the uninfected group (Figure 1, Table 1).

The median IgG_S_ antibody titers declined more rapidly in previously infected study participants compared with uninfected participants (Figure 1, Table 1).

### 3.3. Antibody Response to Vaccine in Previously Infected Participants

In previously infected participants, the median IgG_S_ titer had decreased by 73.42% at 3 months, by 90.80% at 6 months, and by 92.55% at 7 months (Figure 1, Table 1).

No significant differences in circulating IgG_S_ antibody titers were observed between previously infected females and males (Table 2).

However, significantly higher median IgG_S_ antibody titers at the first six testing time points were detected in previously infected study participants aged 45–66 years compared with previously infected individuals aged 24–44 years (*p* = 0.018, *p* = 0.027, *p* = 0.027, *p* = 0.010, *p* = 0.029, *p* = 0.008, *p* = 0.691, respectively) (Table 2).

### 3.4. Antibody Response to Vaccine in Uninfected Participants

Among the uninfected individuals, data analysis showed that the median IgG_S_ titer had decreased by 69.21% at 3 months, by 89.62% at 6 months, and by 92.46% at 7 months (Figure 1, Table 1).

No significant differences in circulating IgG_S_ antibody titers between females and males nor between age groups were observed (Table 2).

### 3.5. Comparison of IgG_S_ Antibody Response to Vaccine between Previously Infected and Uninfected Participants According to Gender and Age Group

The median titer of IgG_S_ was significantly higher in previously infected individuals compared with previously uninfected subjects, at all seven time points after complete vaccination (*p* < 0.001 for each of the seven time points, respectively) (Table 1).

Statistical analysis by gender showed a more rapid decline in antibody titer in previously infected males compared with uninfected males, while in females the decline trend was similar between previously infected and uninfected individuals.

**Figure 2:**
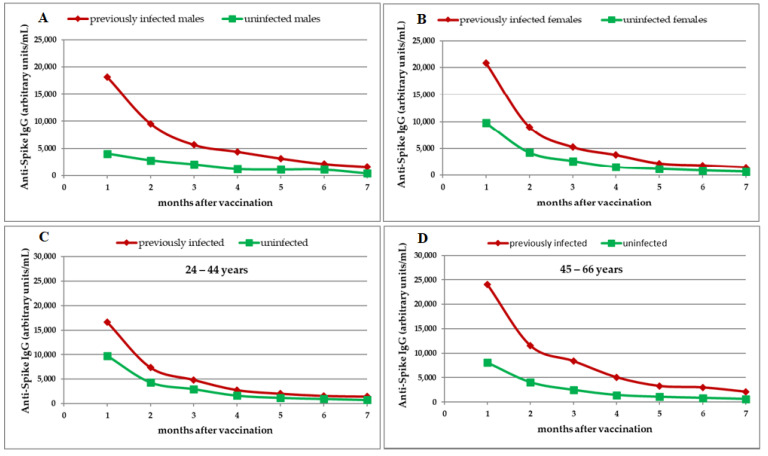
Dynamics of SARS-CoV-2 anti-spike (S) protein IgG (IgG_S_) antibodies after complete vaccination in previously infected and uninfected study participants. Antibody titers were evaluated monthly for 7 months. Median titers of IgG_S_ antibodies in males (**A**), females (**B**), healthcare workers aged 24–44 years (**C**), and healthcare workers aged 45–66 years (**D**).

In previously infected males, the median IgG_S_ titer had decreased by 68.71% at 3 months, by 88.28% at 6 months, and by 91.22% at 7 months, compared with uninfected males where the median IgG_S_ titer had decreased by 49.41% at 3 months, by 71.58% at 6 months, and by 89.59% at 7 months (Table 2, Figure 2A).

In previously infected females, the median IgG_S_ titer had decreased by 74.83% at 3 months, by 91.08% at 6 months, and by 92.79% at 7 months. A similar trend was seen in uninfected females: the median IgG_S_ titer had decreased by 72.87% at 3 months, by 91.22% at 6 months, and by 93.32% at 7 months (Table 2, Figure 2B).

The rate of IgG_s_ decline was similar in previously infected and uninfected individuals aged 24–44 years and in individuals aged 45–66 years (Table 2, Figure 2C,D).

Our results revealed no significant difference in median IgG_S_ antibody titers between previously infected individuals aged 24–44 years and those previously uninfected from the same age group (Table 2). Significantly higher median IgG_S_ antibody titers at all seven time points were detected in previously infected individuals aged 45–66 years compared with those previously uninfected from the same age group (*p* < 0.001 for each of the seven time points, respectively) (Table 2).

## 4. Discussion

We evaluated on a monthly basis the dynamics of SARS-CoV-2 anti-spike (S) protein IgG_S_ in Romanian healthcare worker vaccinees with and without SARS-CoV-2 past infection. Our results indicate a significant decrease in the median IgG_S_ antibody titers at 7 months after complete vaccination in both SARS-CoV-2 previously infected and uninfected individuals. The waning of humoral response after administration of the second dose of the BNT162b2 vaccine has been recently reported by other authors [7,12]. Remarkably, we noticed the persistence of IgG_S_ antibodies throughout the 7 months after the second dose of the vaccine in all study participants.

Our data showed that the two-dose regimen of vaccination resulted in significantly higher IgG_S_ antibody titers in individuals with previous SARS-CoV-2 natural infection compared with infection-naïve subjects, in agreement with other recent studies [9,13,14,15]. In response to SARS-CoV-2 infection, humans generate specific antibodies, CD4^+^ T cells and CD8^+^ T cells, which leads to a substantial immune memory [16]. Specifically, after SARS-CoV-2 infection, antibodies for the spike (S) glycoprotein are produced and will persist for at least 6 months [17]. This may explain the stronger immune response seen in previously infected individuals compared with uninfected individuals

In the infected group of participants, no significant differences in circulating IgG_S_ antibody titers were observed between females and males. Interesting, previously infected individuals aged 45–66 years had a significantly stronger immune response compared with infected individuals aged 24–44 years. Recent studies have shown that older adults may develop a stronger immune response against SARS-CoV-2 compared with younger adults [18,19]. It has been suggested that most vaccinations (e.g., for influenza or tuberculosis) in older adults are to boost pre-existing immunity from natural infections (including coronaviruses) and previous vaccinations [20,21], and this may explain the significantly higher antibody response in the 45–66 years age group compared with those aged 24–44 years.

Although higher median IgG_S_ antibody titers at all seven time points were detected in previously infected individuals aged 45–66 years compared with previously uninfected individuals from the same age group, no significant differences were observed between previously infected and uninfected individuals aged 24–44 years.

The small number of participants in our study may be considered a limitation. In addition, participants were healthcare workers without any known medical pathology, and our cohort did not include immunocompromised people or individuals aged >66 years.

This study brings new and important information regarding the dynamics of antibody response during a 7-month period after administration of the BNT162b2 mRNA COVID-19 vaccine. Assessment of antibody response to the vaccine was carried out according to gender and age groups in both SARS-CoV-2 previously infected and uninfected individuals. The results of the present study may represent a starting point for further scientific research, including monthly assessment of antibody response for at least 1 year after vaccination. Also, further studies are needed to ascertain the antibody response in older individuals and in immunocompromised people.

## 5. Conclusions

The results of the present study indicate a significant decrease in the median IgG_S_ antibody titers at 7 months after complete vaccination with the BNT162b2 mRNA COVID-19 vaccine in both SARS-CoV-2 previously infected and uninfected individuals. Our data reveal that full vaccination resulted in significantly higher IgG_S_ antibody titers in SARS-CoV-2 previously infected individuals compared with previously uninfected subjects. Although the IgG_S_ antibody titers declined every month after vaccination, our results indicate the persistence of antibody response in all study participants at 7 months. Further studies should be conducted to monitor the antibody response to the BNT162b2 mRNA COVID-19 vaccine (Pfizer-BioNTech) beyond 7 months and to assess the need for a new booster dose in order to extend the duration and amplitude of the specific immune response, taking into account the current Delta variant surge.

## Figures and Tables

**Table 1:** Median and interquartile range of SARS-CoV-2 IgG_S_ antibody test results in study participants with or without previous SARS-CoV-2 infection. Circulating levels of SARS-CoV-2 anti-spike (S) protein IgG (IgG_S_) antibodies in serum samples were evaluated monthly for 7 months after the second dose of the vaccine (Pfizer-BioNTech, BNT162b2 mRNA COVID-19).

Time after Second Vaccine Dose	Previously Infected Participants	Uninfected Participants	*p*-Value
1 month	20,160.0 (8572.4; 32,247.2)	8386.2 (4015.3; 13,374.1)	<0.001
2 months	9196.4 (4197.1; 21,490.9)	4159.7 (2170.9; 6205.3)	<0.001
3 months	5357.6 (2830.2; 9806.7)	2582.2 (1285.3; 4692.4)	<0.001
4 months	3837.8 (2100.1; 9051.9)	1459 (785.9; 2789.9)	<0.001
5 months	2442.5 (1678; 5282.0)	1160.3 (591.5; 1990.9)	<0.001
6 months	1855.7 (1234.3; 3462.6)	870.7 (480.8; 1566.1)	<0.001
7 months	1501.4 (916.4; 2743.0)	632.4 (385; 1151.8)	<0.001

**Table 2:** Median and interquartile range of SARS-CoV-2 IgG_S_ antibody test results in previously infected and uninfected study participants according to gender and age group. Circulating levels of SARS-CoV-2 anti-spike (S) protein IgG (IgG_S_) antibodies in serum samples were evaluated monthly, for 7 months, after the second dose of the vaccine (Pfizer-BioNTech, BNT162b2 mRNA COVID-19).

Time after 2nd Vaccine Dose	SARS-CoV-2 Anti-Spike IgG Antibody Test Results (AU/mL): Median (Interquartile Range)							
	Gender				Age Groups			
	Male		Female		24–44 Years		45–66 Years	
	Previously Infected Participants	Uninfected Participants	Previously Infected Participants	Uninfected Participants	Previously Infected Participants	Uninfected Participants	Previously Infected Participants	Uninfected Participants
1 month	18,110.0 (7823.8; 29,370.7)	4015.3 (2967.4; 16,862.6)	20,812.6 (8572.4; 34,575.2)	9750.9 (6087.4; 13,231.3)	16,629.7 (5365.5; 20,812.6)	9760.3 (5587.7; 15,278.1)	24,014.4 (9492.35; 40,000)	8069.0 (3663.55; 13,169.9)
2 months	9506.6 (4043.95; 21,714.6)	2781.7 (1563.9; 7177.8)	9005.5 (4473.1; 20,102.3)	4221.1 (2782.9; 6169.2)	7377.0 (3248.2; 9701.9)	4314.5 (2660.7; 7585.7)	11,520.4 (5113.8; 27,542.9)	4053.4 (2151.5; 6205.3)
3 months	5667.1 (2776.45; 12,044.0)	2031.4 (661.5; 4974.0)	5239.1 (3503.2; 9763.8)	2645.0 (1747.2; 4262.6)	4865.8 (1812; 5976.7)	2953.7 (1500.3; 4899.0)	8388.1 (3402.5; 21,722.95)	2485.0 (1171.4; 4410.2)
4 months	4398.2 (2100.1; 9182.6)	1240.8 (535.9; 4168.4)	3772.0 (2089.7; 8541.7)	1518.7 (897.25; 2648.1)	2794.5 (1085.9; 3816.2)	1625.6 (802.6; 3196.2)	5083.1 (2414.4; 15,228.4)	1439.8 (720.4; 2714.7)
5 months	3144.2 (1225.35; 7221.5)	1154.4 (429.1; 3114.7)	2212.1 (1826.1; 5282.0)	1162.1 (594.9; 1798.3)	2063.8 (1084.2; 2488.1)	1171.4 (592.2; 1924.1)	3291.7 (1796.9; 14,634.9)	1062.0 (590.8; 2110.3)
6 months	2122.3 (1131.6; 3277.4)	1140.9 (376.25; 2520.55)	1855.7 (1521.2; 5517.7)	856.5 (588.4; 1256.4)	1614.5 (533.8; 2535.2)	955.9 (477.95; 1481.5)	3005.2 (1615.3; 12,451.0)	836.3 (480.8; 1566.1)
7 months	1590.2 (916.4; 2875.5)	418.0 (189.3; 1160.6)	1501.4 (1131.8; 2668.1)	651.3 (440.2; 1151.8)	1468.2 (724.2; 2119.1)	737.3 (397.7; 1971.1)	2126.3 (1071.1; 5328.0)	618.2 (311.3; 1044.0)
